# 

**Published:** 1869-06-15

**Authors:** 


### *CONTENTS.*

REPORTS.

American Medical Association—Session for 1869 ........ 365

COMMUNICATIONS.

Philadelphia Letter ........ 373

Lacerated and Contused Wound—Treatment of. By C. F. Hart, M. D., Chicago ........ 375

Super Oxygenation, a Reply to Criticism and Strictures. By S. S. Wallihan, M. D., Chicago ........ 380

Evanston Letter ........ 384

EDITORIAL.

Publication of the Journal ........ 384

Microscopy ........ 385

New Journal ........ 387

Medical Journalists ........ 387

MISCELLANY.

Chemistry of the Cinchona Barks.—Cincho-Quinine ........ 389

The Infection of Crime ........ 393

Prolonged Anuria ........ 395

The Physiological Action of Picrotoxin ........ 395

A Caution to Prescribers ........ 396

Brainard Medical Society ........ 396

